# Palliative radiotherapy in patients with a poor performance status: the palliative effect is correlated with prolongation of the survival time

**DOI:** 10.1186/1748-717X-8-166

**Published:** 2013-07-05

**Authors:** Shinsaku Yamaguchi, Takayuki Ohguri, Yuichi Matsuki, Katsuya Yahara, Hiroyuki Narisada, Hajime Imada, Yukunori Korogi

**Affiliations:** 1 Department of Radiology, Kitakyushu General Hospital, Kitakyushu, Japan; 2 Department of Radiology, University of Occupational and Environmental Health, Kitakyushu, Japan; 3 Department of Cancer therapy center, Tobata Kyoritsu Hospital, Kitakyushu, Japan

**Keywords:** Palliative radiotherapy, Symptomatic relief, Support team assessment schedule

## Abstract

**Background:**

The purpose of this study was to analyze the efficacy and tolerability of palliative radiotherapy (RT) in patients with a poor performance status (PS) and to evaluate the relationship between the palliative effect and survival time.

**Methods:**

One hundred and thirty-three patients with a poor PS (Eastern Cooperative Oncology Group 3 or 4) were treated with palliative RT using the three-dimensional conformal technique and retrospectively analyzed. Each patient's primary symptom treated with palliative RT as the major cause of the poor PS was evaluated using the second item of the Support Team Assessment Schedule (STAS) at the start and one week after the completion of palliative RT.

**Results:**

One hundred and fourteen (86%) of the 133 patients completed the planned palliative radiation dose. Grade 3 acute toxicity was observed in two patients (2%) and Grade 2 acute toxicity was observed in 10 patients (9%). No Grade 2 or higher late toxicities were observed, except for Grade 3 radiation pneumonitis in one patient. Improvement in the STAS scores between pre- and post-palliative RT was recorded in 76 (61%) of the 125 patients with available scores of STAS. A significant improvement in the mean STAS score between pre- and post-palliative RT was recognized (*p* < 0.0001). Improvement in the STAS score was found to be the most statistically significant prognostic factor for overall survival after palliative RT in both the multivariate and univariate analyses. The median overall survival time in the patients with an improvement in the STAS score was 6.4 months, while that in the patients without improvement was 2.4 months (*p* < 0.0005).

**Conclusions:**

Palliative RT in patients with a poor PS provides symptomatic benefits in more than half of patients without inducing severe toxicities. The palliative effect is strongly correlated with prolongation of the survival time and may contribute to improving the remaining survival time in patients with metastatic/advanced cancer with a poor PS.

## Background

Radiation therapy (RT) is widely used to treat cancer, which has become a leading cause of death in Japan, with 275,000 Japanese patients dying of cancer in 2010. RT with palliative intent is administered in approximately 35-50% of cancer patients [1]. Distressing symptoms, including pain, bleeding and obstruction, can often be relieved with minimal toxic effects [2]. Painful bone metastasis, in particular, is common in oncologic practice.

The performance status (PS) has been demonstrated to be strongly correlated with survival as well as treatment outcomes in all types of cancer patients [3]. This may explain why a poor PS is negatively correlated with the RT referral rates [4]. Potential risks, such as serious acute side effects, discomfort associated with the waiting time for treatment, transportation, hospitalization and the duration of the RT course, are concerns in patients with a poor PS [5,6]. Few clinical studies have assessed the effects of palliative RT in patients with a poor PS, and the merit and prognostic factors of palliative RT have not been established [7,8].

Several previous studies have demonstrated that the baseline QoL is a significant predictor of survival in patients with advanced cancer [9-11]. Improvements in the QoL in addition to palliative effects have also been recognized following the administration of palliative RT in several studies [12,13]. The use of palliative RT, especially that administered to treat bone metastasis, should be considered due to its valuable effects, even in patients with an estimated survival time of three months [14]. Several surgical interventions performed with a palliative intent have exhibited survival benefits in advanced cancer patients with a poor PS [15,16]. Poststenting RT effectively prolonged the duration of dysphagia relief and improved the overall survival in patients with inoperable esophageal cancer in a randomized trial [17]. In this context, we hypothesized that a meaningful effect of palliative RT would result in prolongation of the limited survival time in patients with advanced/metastatic cancer with a poor PS.

The Support Team Assessment Schedule (STAS) is a proxy assessment scale used to evaluate palliative care that was originally developed in the UK and is currently used worldwide [18-20]. The second item of the STAS, symptom control, evaluates the patient’s most severe symptom. The reliability of assessing the primary symptom using the second item of the STAS has been previously established [21]. The purpose of this study was to evaluate the palliative effect, assessed based on the second item of the STAS, and tolerability of palliative RT in patients with a poor PS and to analyze the relationship between the palliative effect and the survival time.

## Methods

### Patients

Between May 2006 and May 2012, 350 consecutive patients were treated with palliative RT at the authors’ institution. During the same period, there were 133 consecutive patients with 150 lesions treated with palliative RT with a poor PS (Eastern Cooperative Oncology Group 3 or 4) at the start of palliative RT; these patients were enrolled in this retrospective study. Table 1 shows the patients’ clinical characteristics. The pretreatment evaluation included a complete history, physical examination, complete blood count, body computed tomography scan and, in some cases, ^18^ F-FDG PET/CT and/or magnetic resonance imaging and/or bone scintigraphy. Concomitant diseases associated with a worse performance status were recognized in 19 patients as follows: dementia (n = 8), cerebrovascular disease (n = 5) and others (n = 6). This study was approved by the Institutional Review Board of the authors’ institution.

**Table 1:** Patient clinical characteristics

**Variable**	**n (%)**
Age (y)	
Median (range)	75 (38-100)
Gender	
Male	79 (59)
Female	54 (41)
PS	
3	103 (77)
4	30 (23)
Concomitant disease associated with worsening PS	
Yes	19 (14)
No	114 (86)
Primary site	
Lung	51 (38)
Stomach	8 (6)
Esophagus	8 (6)
Bladder	8 (6)
Breast	7 (5)
Colorectum	7 (5)
Prostate	7 (5)
Liver	6 (5)
Other	31 (23)
No. of metastatic lesion	
0-3	30 (23)
≧ 4	103 (77)
Histology	
Squamous cell carcinoma	30 (23)
Adenocarcinoma	35 (26)
Small cell carcinoma	15 (11)
Hepatocellular carcinoma	6 (5)
Transitional cell carcinoma	6 (5)
Other	41 (31)
No. of irradiated site	
One site	117 (88)
Two sites	15 (11)
Three sites	1 (1)
Irradiated site (n = 150)	
Primary tumor	
Bladder	7 (5)
Lung	7 (5)
Esophagus	5 (3)
Uterus	3 (2)
Rectum	3 (2)
Others	9 (6)
Metastatic tumor	
Bone	86 (57)
Vertebrate	47
Pelvic	17
Rib	10
Femur	4
Others	8
Brain	18 (12)
Mediastinum	5 (3)
Head and neck	3 (2)
Others	4 (3)

### Palliative radiotherapy

All patients were treated with external RT using a linear accelerator with 4, 10 MV or 5–15 MeV electrons (Toshiba PRIMUS linear accelerator equipped with standard multi-leaf collimators). Computed tomography (CT) images were obtained in 5-mm increments over the region of interest. Three-dimensional conformal RT was performed using the Xio (CMS Japan, Tokyo, Japan) treatment planning system in all patients. The clinical target volume (CTV) was defined as the primary tumor or metastatic site (gross tumor volume) plus a 0.5-1.0-cm margin. The planned target volume (PTV) was defined as the CTV plus 0.5-1.0 cm for the daily setup variation and respiratory movement. To reduce the irradiation dose to the organs at risk, 34 (23%) of the 150 lesions were treated with a three- or four-field beam arrangement or conformational therapy. The schedule of palliative RT was as follows: 30 Gy in 10 fractions in 95 lesions (63%), 20 Gy in five fractions in seven lesions (5%), 39 Gy in 13 fractions in five lesions (3%), 8 Gy in one fraction in two lesions (1%) and other in 41 lesions (27%).

### STAS measurements

Each patient's primary symptom treated with palliative RT as the major cause of the poor PS was evaluated using the second item of the STAS (Japanese version) at the start of and one week after the completion of palliative RT by one of four radiation oncologists. The second item of the STAS, the various symptom control item, was rated on a 5-point Likert scale with the following definitions: 0 = none, 1 = occasional, single or few symptom(s), the patient performs usual activities and is not bothered by the symptom(s), 2 = moderate distress, occasional bad days, the symptoms limit some activities depending on the extent of the disease, 3 = severe symptom(s) present often, activities and concentration are markedly affected by the symptom(s), 4 = severe and continuous overwhelming symptom(s), the patient is unable to think of other matters.

### Evaluation of the tumor response, progression criteria and toxicity

The tumor response was evaluated according to the Response Evaluation Criteria in Solid Tumors using CT and/or a physical examination [22]. The absence of local progression was defined as locally controlled disease, and local control was analyzed in patients monitored for a minimum follow-up of one month. The overall survival was calculated from the first day of palliative RT to the date of death.

Toxicity was graded using the National Cancer Institute Common Toxicity Criteria (CTCAE) version 3. The highest toxicity grade for each patient was used for the toxicity analysis. The toxicity was defined as acute (occurring during therapy and up to three months after therapy) or late (over three months after the completion of therapy).

### Statistical analysis

The Wilcoxon signed-rank test was used to assess the STAS scores between pre- and postpalliative RT. Univariate analyses using Fisher’s exact test and multivariate analyses using logistic regression were performed to evaluate the effects of certain factors on improvement of the STAS score. The overall survival and local control rates were calculated using the Kaplan–Meier method. The statistical significance of the differences between the actuarial curves was assessed using the log-rank test. To identify prognostic factors for overall survival, univariate analyses were performed using gender, age, PS, concomitant disease, number of tumors, irradiated site, total RT dose, completion of the planned RT dose, chemotherapy, primary tumor site, target of palliative RT and improvement in the STAS score. Multivariate analyses using the Cox proportional–hazards model were performed to determine the overall survival rates with respect to the PS, concomitant disease, number of tumors, total RT dose, completion of the planned RT dose, primary tumor site and improvement in the STAS score.

## Results

The median follow-up period was 2.3 months (range 0.0–47.4) in all patients. One hundred and fourteen (86%) of the 133 patients completed the planned palliative RT dose. The remaining 19 patients (14%) were unable to complete the planned dose due to the following reasons: worsening of the patient’s general condition/comorbidities in 14 patients, patient refusal to continue palliative RT in four patients and acute radiation proctitis in one patient. Regarding acute toxicities, Grade 3 complications were observed in two patients (appetite loss and proctitis) and Grade 2 complications were observed in 10 patients (esophagitis in six patients, appetite loss in two patients, oral mucositis in one patient and tinnitus in one patient). With respect to late toxicities, there were no Grade 2 or higher complications, except for Grade 3 radiation pneumonitis in one patient with lung cancer.

**Figure 1:**
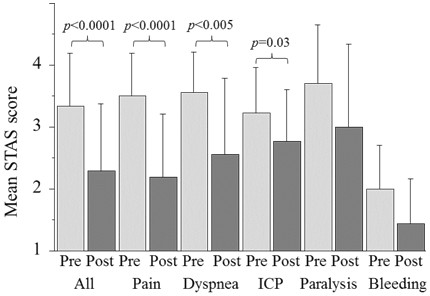
**Mean STAS scores pre- and postpalliative radiotherapy.** ICP: increased intracranial pressure.

The STAS scores of eight (6%) of the 133 patients were not evaluated due to dementia caused by brain metastases in five patients and deterioration of consciousness in three patients. Improvements in the STAS scores between pre- and postpalliative RT were recorded in 76 (61%) of the total 125 patients, 35 (64%) of the 54 patients with pain, nine (64%) of the 14 patients with dyspnea, six (46%) of the 13 patients with symptoms of increased intracranial pressure, three (30%) of the 10 patients with paralysis and four (44%) of the nine patients with bleeding. The mean STAS scores of all patients at pre- and postpalliative RT were 3.3 and 2.3, respectively, with a statistically significant difference (*p* < 0.0001) (Figure 1). The mean STAS scores at pre- and postpalliative RT were 3.6 and 2.2 (*p* < 0.0001) in the 54 patients with pain, 3.6 and 2.6 (*p* = 0.005) in the 14 patients with dyspnea, 3.2 and 2.8 (*p* = 0.03) in the 13 patients with symptoms of increased intracranial pressure, 3.7 and 3.0 (*p* = 0.09) in the 10 patients with paralysis and 2.0 and 1.4 (*p* = 0.05) in the nine patients with bleeding, respectively (Figure 1). Table 2 shows the results of the univariate and multivariate analyses performed to evaluate the effects of certain factors on improvements in the STAS scores among the 125 patients with available STAS scores. According to the univariate analyses, the irradiated site of bone, completion of the planned RT dose and a total RT dose of ≧30 Gy were found to be significant factors. According to the multivariate analyses, the irradiated site of bone and a performance grade of 3 were found to be significant factors.

**Table 2:** Univariate and multivariate analyses to evaluate effects of certain factors on an improvement of STAS score in 125 patients with available scores of STAS

		**Univariate**	**Multivariate**
	**n**	***p***	***p***
Gender			
M/F	76/49	0.85	-
Age			
< 70/≧ 70	47/78	0.56	-
Performance status			
3/4	98/27	0.074	0.013
Concomitant disease			
Yes/No	14/111	0.078	0.322
No. of tumor lesion			
1-3/≧ 4	25/100	0.36	-
Irradiated site			
Bone/others	71/54	0.0054	0.0074
Target of palliative RT			
Metastatic/primary	97/28	0.20	-
Primary tumor site			
Lung/others	51/74	0.58	-
Total RT dose (Gy)			
< 30/≧ 30	20/105	0.047	0.78
Completion of planned RT dose			
Yes/No	109/16	0.013	0.16
Chemotherapy			
Yes/No	25/100	0.82	-

*RT* radiotherapy.

**Figure 2:**
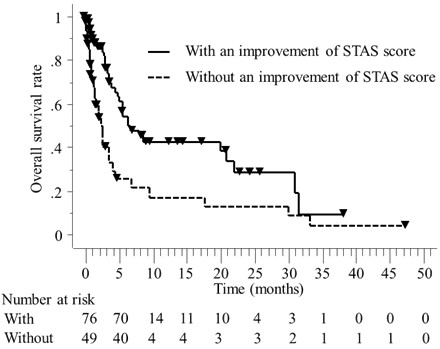
**An improvement in the STAS score between pre- and postpalliative RT was found to be a statistically significant prognostic factor for overall survival after palliative RT (*p*** **= 0.0005).**

The median overall survival time of all patients was 4.5 months. The overall survival rate at one year in all patients was 31%. The 1-year local control rate for the 93 patients who were monitored for a minimum follow-up of one month was 72%. In the univariate analyses of the 125 patients with available STAS scores, the statistically significant factors for better overall survival rates were as follows: an improvement in the STAS score, the presence of concomitant diseases, completion of the planned RT dose, a non-lung primary tumor site, a performance status of 3 and the presence of one to three tumors (Figure 2). The median overall survival time in the patients with an improvement in the STAS score was 6.4 months, while that of the patients without an improvement of the STAS score was 2.4 months (*p* = 0.0005) (Figure 2). In the multivariate analyses of the 125 patients, an improvement in the STAS score, the presence of concomitant diseases, a PS of 3 and a non-lung primary tumor site were found to be statistically significant prognostic factors (Table 3). An improvement in the STAS score was the most statistically significant prognostic factor for overall survival following palliative RT in both the multivariate and univariate analyses.

**Table 3:** Results of the univariate and multivariate analyses of certain factors for overall survival rates after palliative RT in125 patients with available scores of STAS

		**Univariate**		**Multivariate**	
		**MST**		**OR**	
	**n**	**(mos)**	***p***	**(95% CI)**	***p***
Gender					
M	76	4.5	0.20	-	-
F	49	6.3			
Age					
< 70	47	5.6	0.65	-	-
≧ 70	78	4.1			
Performance status					
3	98	6.3	0.026	1.9	0.064
4	27	2.0		(1.0-3.9)	
Concomitant disease*					
Yes	14	26.8	0.011	4.5	0.0084
No	111	3.8		(1.5-13.7)	
No. of tumor lesion					
1-3	25	6.3	0.041	1.4	0.42
≧ 4	100	3.6		(0.6-3.1)	
Irradiated site					
Bone	71	4.7	0.94	-	-
Others	54	4.4			
Target of palliative RT					
Metastatic tumor	97	3.8	0.14	-	-
Primary tumor	28	6.3			
Primary tumor site					
Lung	51	3.4	0.020	2.2	0.011
Others	74	6.1		(1.2-3.9)	
Total RT dose (Gy)					
<30	20	0.7	0.080	1.1	0.91
≧ 30	105	4.9		(0.4-2.7)	
Completion of planned RT dose					
Yes	109	5.0	0.0025	2.0	0.170
No	16	0.5		(0.7-5.2)	
Chemotherapy					
Yes	25	5.6	0.82	-	-
No	100	3.9			
Improvement of STAS score					
Yes	76	6.4	0.0005	3.7	<0.0001
No	49	2.4		(2.0-6.6)	

*RT* radiotherapy, *STAS* Support Team Assesment Schedule, *MST* median survival time, *OR* odds ratio, *CI* confidence interval.

*associated with a worse performance status.

## Discussion

The present study is the first study to assess the various palliative RT effects observed in patients with a poor PS using the second item of the STAS. To our knowledge, previous reports of detailed treatment results of palliative RT in patients with a poor PS are limited [7,8]. Yamazaki et al. analyzed the feasibility of palliative RT in patients with an ECOG PS of 3–4 and found that the treatment completion rate (79%) in the patients with a PS of 3–4 was significantly worse than that (89%) observed in the patients with a PS of 0–2 [8]. Campos et al. demonstrated that the PS, as measured by KPS, is significantly related to the response rate one month after palliative RT in patients with painful bone metastases. In their study, the response rate was 44% in patients with a KPS of ≦40, 44% in patients with a KPS of 50–60, 57% in patients with a KPS of 70–80 and 68% in patients with a KPS of 90–100; however, no such relationships were observed at two or three months [7]. Dennis et al. reported that patients suffering from painful bone metastases with an estimated survival of three months whose median KPS score is 60 should still be considered for palliative RT because, in their study, the overall rate of pain relief was as favorable as 70% at one month and 63% at two months [14]. Similarly, in the current study, the treatment completion rate was 86%, and an improvement in the STAS score following palliative RT was recorded in 64% of the patients with pain. In addition, a PS of 3 was found to be a better predictive factor of improvements in the STAS score than a PS of 4 in the multivariate analyses. We confirmed that administering palliative RT in patients with a poor PS is feasible and offers significant palliative effects, especially in patients with pain, dyspnea and symptoms of increased intracranial pressure.

Previous studies have shown that the baseline QoL is a strong independent predictor of survival in patients with advanced cancer [9-11]. Langendijk et al. reported that the global QoL assessed using the EORTC QLQ-C30 is also a strong prognostic factor for survival in patients with NSCLC treated with RT [23]. There are few reports regarding the correlation between the palliative effects of RT and the duration of survival [24]. Stevens et al. reported that, according to a multivariate analysis, the independent predictive factors for overall survival in head and neck cancer patients treated with palliative RT included a positive treatment response and a higher radiation dose [24]. Recently, Temel et al. examined the effects of introducing palliative care early after diagnosis in a randomized trial of patients with metastatic non-small cell lung cancer and demonstrated that providing early palliative care led to significant improvements in both the quality of life and duration of survival [25]. Nieder et al. demonstrated that, in a randomized clinical trial of whole-brain RT versus best supportive care in patients with brain metastases and adverse prognostic factors, significant improvements in survival time were observed in the 30 Gy whole-brain RT group in comparison to the 20 Gy whole-brain RT group [26]. In the current study, an improvement in the STAS score following palliative RT was found to be the most statistically significant prognostic factor for overall survival in both the univariate and multivariate analyses. Therefore, we suppose that the good palliative effects of RT observed in patients with a poor PS can result in not only improvement of the QoL, but also prolongation of the limited survival time.

Chow et al. reported a predictive model for survival from the time of presentation in an outpatient palliative RT clinic and found that six prognostic factors (primary cancer site, site of metastases, Karnofsky PS and the fatigue, appetite and shortness-of-breath items on the Edmonton Symptom Assessment Scale) had a statistically significant impact in the 395 patients who were prospectively analyzed [27]. In the current study, the palliative effect at one week after the completion of RT was found to be the most statistically significant prognostic factor for overall survival, although the primary cancer site and PS were also identified as significant predictors. Therefore, the palliative RT effect, as well as previously reported prognostic factors, may contribute to predicting the survival time in metastatic/advanced cancer patients with a poor PS.

The proxy assessment of the STAS scale has been used in various palliative settings, including support teams in the community, hospitals and hospices and in both cancer and acquired immunodeficiency syndrome patients [18-21]. Patient self-assessment of symptoms is the gold standard in the palliative care setting. However, patients with a poor PS are unstable and suffer from various symptoms; therefore, completing a self-assessment of symptoms is difficult for these patients. In such situations, the use of a proxy assessment scale, such as the STAS, by medical practitioners is useful because an improvement in the STAS score was found to be the most statistically significant prognostic factor in the current study.

A meta-analysis of dose-fractionation RT trials found no significant differences in complete or overall pain relief between single- and multifraction palliative RT in patients with bone metastasis [28]. Most cancer patients destined for palliative RT have a very limited life span. In such situations, finding a balance between the potential benefits of treatment and the risk of toxicity and inconvenience for the patient is essential. The use of protracted treatment regimens is not appropriate in patients with a poor PS. In the current study, single-fraction RT was administered in only two patients, and 14% of the patients were unable to complete the planned palliative RT dose primarily due to general deterioration. Therefore, we suggest that single-fraction palliative RT be considered in such cases and may be worthwhile, especially in patients with poor prognostic factors for survival.

Our study is associated with several limitations. First, it is a retrospective study that introduces the possibility of treatment selection bias. Moreover, it was a relatively small case series conducted in a single institute, and both the patient profiles and RT regimens were heterogeneous. Therefore, in the future, large prospective studies are required to investigate the actual benefits of palliative RT, including the effects on survival and the QoL, in patients with a poor PS.

## Conclusions

In summary, this is the first report that attempted to assess the efficacy and tolerability of palliative RT in patients with a poor PS and to evaluate the relationship between the palliative RT effect and the survival time using the second item of the STAS. The use of palliative RT in patients with a poor PS was found to be feasible, and significant palliative effects were obtained, especially for pain, dyspnea and symptoms of increased intracranial pressure. The palliative effect at one week after the completion of RT was found to be strongly correlated with prolongation of the survival time, which may contribute to the assessment of the remaining survival time in metastatic/advanced cancer patients with a poor PS. These results justify conducting further prospective evaluations with detailed treatment protocols, including those regarding the radiation dose and fractionation, to clarify whether palliative RT can improve the limited survival time and QoL in patients with a poor PS.

## Abbreviations

RT: Radiotherapy; PS: Performance status; STAS: Support team assessment schedule

## Competing interests

The authors declare that they have no competing interests.

## Authors’ contributions

SY collected and analyzed data, and drafted the manuscript; TO analyzed data and drafted the manuscript; YM collected and analyzed data; KY contributed to data analysis; HN contributed to data analysis; HI contributed to data analysis; YK revised the manuscript. All authors read and approved the final manuscript.

## References

[B1] Cancer pain relief and palliative care Report of a WHO expert committee World Health Organ Tech Rep Ser 1990 804 1 75 1702248

[B2] Hoegler D Radiotherapy for palliation of symptoms in incurable cancer Curr Probl Cancer 1997 21 129 183 10.1016/S0147-0272(97)80004-9 9202888

[B3] Glare PA Sinclair CT Palliative medicine review: prognostication J Palliat Med 2008 11 84 103 10.1089/jpm.2008.9992 18370898

[B4] Huang J Zhou S Groome P Tyldesley S Zhang-Solomans J Mackillop WJ Factors affecting the use of palliative radiotherapy in Ontario J Clin Oncol 2001 19 137 144 11134206 10.1200/JCO.2001.19.1.137

[B5] Lutz ST Chow EL Hartsell WF Konski AA A review of hypofractionated palliative radiotherapy Cancer 2007 109 1462 1470 10.1002/cncr.22555 17330854

[B6] Lutz S Spence C Chow E Janjan N Connor S Survey on use of palliative radiotherapy in hospice care J Clin Oncol 2004 22 3581 3586 10.1200/JCO.2004.11.151 15337808

[B7] Campos S Presutti R Zhang L Salvo N Hird A Tsao M Barnes EA Danjoux C Sahgal A Mitera G Sinclair E DeAngelis C Nguyen J Napolskikh J Chow E Elderly patients with painful bone metastases should be offered palliative radiotherapy Int J Radiat Oncol Biol Phys 2010 76 1500 1506 10.1016/j.ijrobp.2009.03.019 19540056

[B8] Yamazaki H Inoue T Yoshida K Imai A Yoshioka Y Tanaka E Shimamoto S Nakamura S Yamada Y Nakamura H Araki Y Changes in performance status of elderly patients after radiotherapy Radiat Med 2001 19 9 18 11305622

[B9] Coates A Porzsolt F Osoba D Quality of life in oncology practice: prognostic value of EORTC QLQ-C30 scores in patients with advanced malignancy Eur J Cancer 1997 33 1025 1030 10.1016/S0959-8049(97)00049-X 9376182

[B10] Maisey NR Norman A Watson M Allen MJ Hill ME Cunningham D Baseline quality of life predicts survival in patients with advanced colorectal cancer Eur J Cancer 2002 38 1351 1357 10.1016/S0959-8049(02)00098-9 12091066

[B11] Dancey J Zee B Osoba D Whitehead M Lu F Kaizer L Latreille J Pater JL Quality of life scores: an independent prognostic variable in a general population of cancer patients receiving chemotherapy. the national cancer institute of Canada clinical trials group Qual Life Res 1997 6 151 158 9161115 10.1023/a:1026442201191

[B12] Zeng L Chow E Bedard G Zhang L Fairchild A Vassiliou V Alm El-Din MA Jesus-Garcia R Kumar A Forges F Tseng LM Hou MF Chie WC Bottomley A Quality of life after palliative radiation therapy for patients with painful bone metastases: results of an international study validating the EORTC QLQ-BM22 Int J Radiat Oncol Biol Phys 2012 84 e337 e342 10.1016/j.ijrobp.2012.05.028 22763028

[B13] Caissie A Zeng L Nguyen J Zhang L Jon F Dennis K Holden L Culleton S Koo K Tsao M Barnes E Danjoux C Sahgal A Simmons C Chow E Assessment of health-related quality of life with the European organization for research and treatment of cancer QLQ-C15-PAL after palliative radiotherapy of bone metastases Clin Oncol (R Coll Radiol) 2012 24 125 133 10.1016/j.clon.2011.08.008 21917431

[B14] Dennis K Wong K Zhang L Culleton S Nguyen J Holden L Jon F Tsao M Danjoux C Barnes E Sahgal A Zeng L Koo K Chow E Palliative radiotherapy for bone metastases in the last 3 months of life: worthwhile or futile? Clin Oncol (R Coll Radiol) 2011 23 709 715 10.1016/j.clon.2011.05.004 21665446

[B15] Amaral L Mendes J Martins P Bernardo L Quintanilha R Santos V Melo AS Survival benefits of palliative gastric cancer resection - a regional center experience Hepatogastroenterology 2012 59 1651 1656 22683985 10.5754/hge10201

[B16] Ouchi K Sugawara T Ono H Fujiya T Kamiyama Y Kakugawa Y Mikuni J Yamanami H Therapeutic significance of palliative operations for gastric cancer for survival and quality of life J Surg Oncol 1998 69 41 44 10.1002/(SICI)1096-9098(199809)69:1<41::AID-JSO8>3.0.CO;2-K 9762890

[B17] Javed A Pal S Dash NR Ahuja V Mohanti BK Vishnubhatla S Sahni P Chattopadhyay TK Palliative stenting with or without radiotherapy for inoperable esophageal carcinoma: a randomized trial J Gastrointest Cancer 2012 43 63 69 10.1007/s12029-010-9206-4 20835926

[B18] Higginson IJ McCarthy M Validity of the support team assessment schedule: do staffs' ratings reflect those made by patients or their families? Palliat Med 1993 7 219 228 10.1177/026921639300700309 7505183

[B19] Lo RS Ding A Chung TK Woo J Prospective study of symptom control in 133 cases of palliative care inpatients in Shatin hospital Palliat Med 1999 13 335 340 10.1191/026921699677451150 10659102

[B20] Carson MG Fitch MI Vachon ML Measuring patient outcomes in palliative care: a reliability and validity study of the support team assessment schedule Palliat Med 2000 14 25 36 10.1191/026921600677786382 10717720

[B21] Miyashita M Yasuda M Baba R Iwase S Teramoto R Nakagawa K Kizawa Y Shima Y Inter-rater reliability of proxy simple symptom assessment scale between physician and nurse: a hospital-based palliative care team setting Eur J Cancer Care (Engl) 2010 19 124 130 10.1111/j.1365-2354.2008.00967.x 19709165

[B22] Therasse P Arbuck SG Eisenhauer EA Wanders J Kaplan RS Rubinstein L Verweij J Van Glabbeke M van Oosterom AT Christian MC Gwyther SG New guidelines to evaluate the response to treatment in solid tumors. European organization for research and treatment of cancer, National Cancer Institute of the United States, National Cancer Institute of Canada J Natl Cancer Inst 2000 92 205 216 10.1093/jnci/92.3.205 10655437

[B23] Langendijk H Aaronson NK de Jong JM ten Velde GP Muller MJ Wouters M The prognostic impact of quality of life assessed with the EORTC QLQ-C30 in inoperable non-small cell lung carcinoma treated with radiotherapy Radiother Oncol 2000 55 19 25 10788684 10.1016/s0167-8140(00)00158-4

[B24] Stevens CM Huang SH Fung S Bayley AJ Cho JB Cummings BJ Dawson LA Hope AJ Kim JJ O'Sullivan B Waldron JN Ringash J Retrospective study of palliative radiotherapy in newly diagnosed head and neck carcinoma Int J Radiat Oncol Biol Phys 2011 81 958 963 10.1016/j.ijrobp.2010.06.055 20950952

[B25] Temel JS Greer JA Muzikansky A Gallagher ER Admane S Jackson VA Dahlin CM Blinderman CD Jacobsen J Pirl WF Billings JA Lynch TJ Early palliative care for patients with metastatic non-small-cell lung cancer N Engl J Med 2010 363 733 742 10.1056/NEJMoa1000678 20818875

[B26] Nieder C Norum J Dalhaug A Aandahl G Pawinski A Radiotherapy versus best supportive care in patients with brain metastases and adverse prognostic factors Clin Exp Metastasis 2013 10.1007/s10585-013-9573-x 23392634

[B27] Chow E Fung K Panzarella T Bezjak A Danjoux C Tannock I A predictive model for survival in metastatic cancer patients attending an outpatient palliative radiotherapy clinic Int J Radiat Oncol Biol Phys 2002 53 1291 1302 10.1016/S0360-3016(02)02832-8 12128132

[B28] Wu JS Wong R Johnston M Bezjak A Whelan T Meta-analysis of dose-fractionation radiotherapy trials for the palliation of painful bone metastases Int J Radiat Oncol Biol Phys 2003 55 594 605 10.1016/S0360-3016(02)04147-0 12573746

